# Comparative Yield of Different Diagnostic Tests for Tuberculosis among People Living with HIV in Western Kenya

**DOI:** 10.1371/journal.pone.0152364

**Published:** 2016-03-29

**Authors:** Joseph S. Cavanaugh, Surbhi Modi, Susan Musau, Kimberly McCarthy, Heather Alexander, Barbara Burmen, Charles M. Heilig, Ray W. Shiraishi, Kevin Cain

**Affiliations:** 1 United States Centers for Disease Control and Prevention, Atlanta, Georgia, United States of America; 2 Kenya Medical Research Institute (KEMRI) Center for Global Health Research, Kisumu, Kenya; 3 United States Centers for Disease Control and Prevention, Kisumu, Kenya; National AIDS Research Institute, INDIA

## Abstract

**Background:**

Diagnosis followed by effective treatment of tuberculosis (TB) reduces transmission and saves lives in persons living with HIV (PLHIV). Sputum smear microscopy is widely used for diagnosis, despite limited sensitivity in PLHIV. Evidence is needed to determine the optimal diagnostic approach for these patients.

**Methods:**

From May 2011 through June 2012, we recruited PLHIV from 15 HIV treatment centers in western Kenya. We collected up to three sputum specimens for Ziehl-Neelsen (ZN) and fluorescence microscopy (FM), GeneXpert MTB/RIF (Xpert), and culture, regardless of symptoms. We calculated the incremental yield of each test, stratifying results by CD4 cell count and specimen type; data were analyzed to account for complex sampling.

**Results:**

From 778 enrolled patients, we identified 88 (11.3%) laboratory-confirmed TB cases. Of the 74 cases who submitted 2 specimens for microscopy and Xpert testing, ZN microscopy identified 25 (33.6%); Xpert identified those plus an additional 18 (incremental yield = 24.4%). Xpert testing of spot specimens identified 48 (57.0%) of 84 cases; whereas Xpert testing of morning specimens identified 50 (66.0%) of 76 cases. Two Xpert tests detected 22/24 (92.0%) TB cases with CD4 counts <100 cells/μL and 30/45 (67.0%) of cases with CD4 counts ≥100 cells/μl.

**Conclusions:**

In PLHIV, Xpert substantially increased diagnostic yield compared to smear microscopy and had the highest yield when used to test morning specimens and specimens from PLHIV with CD4 count <100 cells/μL. TB programs unable to replace smear microscopy with Xpert for all symptomatic PLHIV should consider targeted replacement and using morning specimens.

## Introduction

Tuberculosis (TB) is the leading cause of death for people living with HIV (PLHIV) worldwide, responsible for an estimated 360,000 deaths in 2013.[1] Active screening for TB among PLHIV has been shown to reduce mortality and morbidity, [2, 3] but laboratory confirmation of TB remains challenging in PLHIV.

In resource-limited settings, detection of acid-fast bacilli (AFB) using sputum smear microscopy is still the most widely used diagnostic test for pulmonary TB. As a diagnostic test, however, it does not perform well among PLHIV, with 30%-50% sensitivity compared to liquid culture in research settings and as low as 9% sensitivity in operational settings.[4, 5] Sputum culture is often considered the standard for laboratory confirmation of TB, but requires resources and technical skill not routinely available in many settings. More recently, an automated polymerase chain reaction platform, the Cepheid Xpert MTB/RIF assay (Cepheid, Sunnyvale CA), was developed for rapid diagnosis of TB.[6] In meta-analysis, the pooled sensitivity of this assay for culture-confirmed TB was 61% in PLHIV with sputum smear-negative TB and 97% in PLHIV with sputum smear-positive TB.[7] The World Health Organization (WHO) recommends Xpert MTB/RIF as the initial diagnostic test among PLHIV.[8] Many countries have expanded implementation and use of Xpert MTB/RIF, especially for PLHIV; however, financial and other logistical considerations, as well as inconsistent utilization, have slowed expansion of this technology.

As countries continue to improve strategies for TB case-finding among PLHIV, it would be helpful to know the programmatic utility of sputum microscopy in settings where Xpert MTB/RIF is available, the incremental value of each Xpert MTB/RIF test performed, and the role of sputum culture in programs using Xpert MTB/RIF. Perhaps more importantly, programs require a better understanding of how patient and specimen characteristics influence the yield of these tests, so that they might maximize their utility while minimizing financial and human resource requirements. We conducted a study to characterize the value of various diagnostic tests, including the incremental yield of Xpert MTB/RIF and culture above smear microscopy on sputum, stool, and lymph node aspirate (LNA) specimens collected from PLHIV and stratified findings by specimen type (morning or spot) and CD4 cell count.

## Methods

### Study design and participants

For enrollment, we stratified all 24 public HIV care and treatment facilities in three districts in western Kenya with at least 200 enrolled patients into small (200–1000 patients, n = 14) and large (>1000 patients; n = 10) clinics. The number of sites selected from each stratum was proportional to the size of the stratum and we randomly selected nine small and six large facilities. Between May 2011 and June 2012, we enrolled consecutive, consenting patients over a ten week period at each clinic. Patients were eligible for the study if they were seven years of age or older, had documented HIV infection, had not been enrolled in any HIV care or treatment program in the preceding two years, and had not received TB treatment at any time in the preceding one year.

### Clinical care and specimen collection

We recorded demographic and clinical information at the initial encounter, screening participants for TB symptoms using a standardized questionnaire. All participants were asked to provide one morning and two spot sputum specimens (hereafter referred to as “spot 1” and “spot 2”). Participants from the three largest facilities were also asked to provide a single stool specimen. We asked trained clinicians at those three facilities, plus one additional facility with sufficient capacity, to aspirate subcutaneous lymph nodes in the head or neck region that were greater than one centimeter in diameter.

### Laboratory procedures

**Fig 1:**
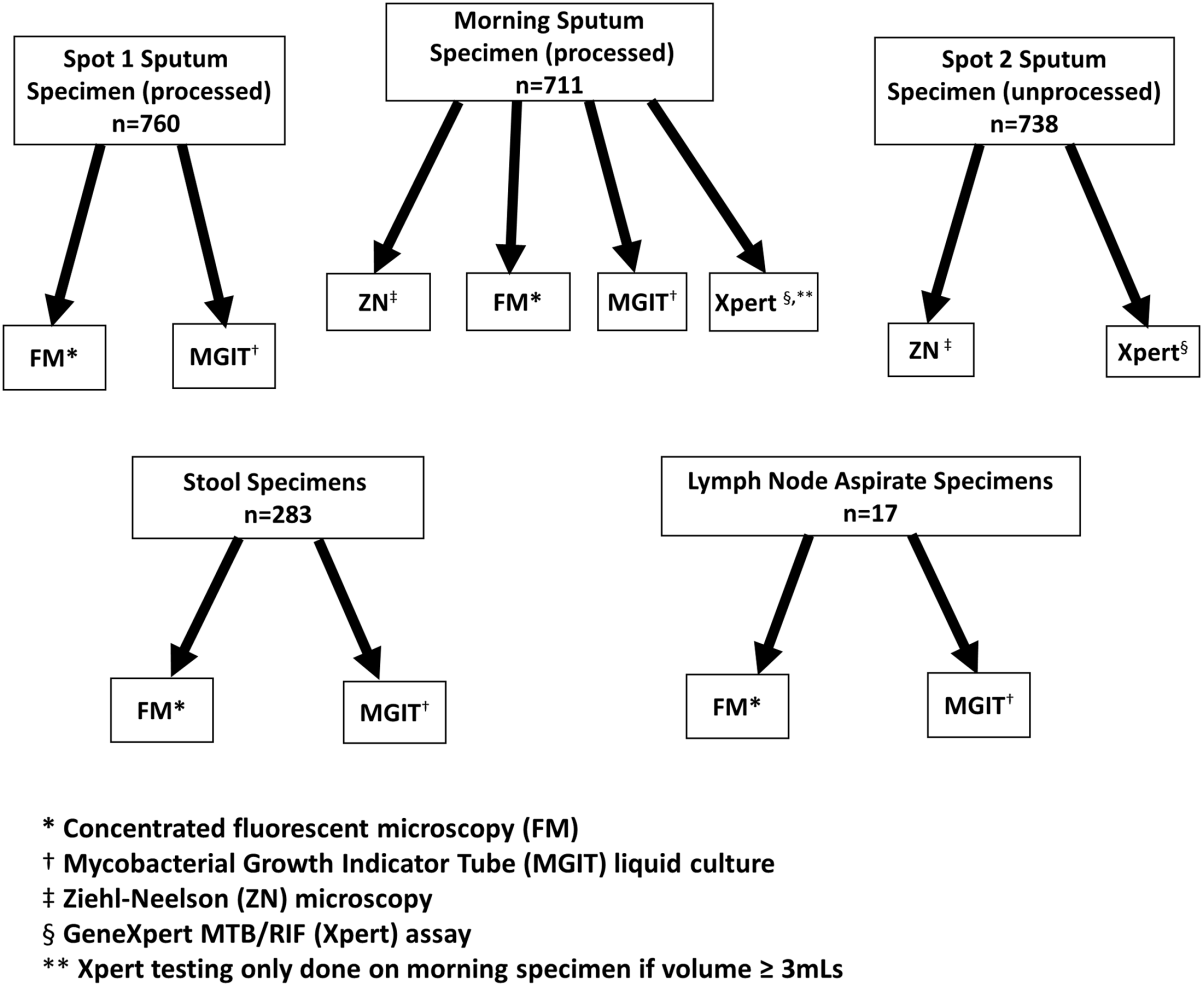
Laboratory tests performed on different specimens.

Specimens were transported to the Kenya Medical Research Institute (KEMRI)/CDC TB reference laboratory in Kisumu for direct Ziehl-Neelsen (ZN) and concentrated fluorescence microscopy (FM), Xpert MTB/RIF testing and mycobacterial culture. Testing algorithms for each specimen are presented in Fig 1.

#### Specimen Processing

We processed spot 1 and morning sputum specimens for culture using standard methods including decontamination with N-acetyl-L-cysteine and sodium hydroxide sodium citrate (1.5% final concentration), followed by centrifugation and resuspension in phosphate buffer (pH 6.8).[9] Because it was not cultured, we did not process spot 2 specimens prior to testing. We processed stool specimens by emulsifying one gram in 10 ml of sterile water, vortexing with sterile glass beads and then filtering through sterile gauze.[10] Lymph node aspirates (LNAs) were not processed prior to culture inoculation.

#### Smear Microscopy

We stained direct smears prepared from the morning and spot 2 sputum specimens according to the ZN method, viewed them under 1000x magnification, and graded them according to World Health Organization (WHO) recommendations.[11] We also prepared direct smears from LNAs with volumes >0.5 ml. We stained LNA smears and the concentrated smears prepared from processed specimen pellets (spot 1, morning sputum, and stool), using FM methods, viewed them under 400x magnification, and graded them according to WHO recommendations.[11] All positive smears were confirmed by a second reader.

#### Xpert^®^ MTB/RIF

We performed Xpert MTB/RIF testing on all spot 2 sputum specimens and on morning sputum specimens with volumes ≥3.0 ml according to manufacturer’s recommendations. Briefly, we mixed sputum specimens with a 2:1 ratio of sample reagent and incubated at room temperature for 15 minutes prior to Xpert MTB/RIF testing.

#### Liquid culture

We cultured stool, LNA, and sputa from spot 1 and morning specimens with the BACTEC Mycobacteria Growth Indicator Tube (MGIT) 960 system [Becton Dickinson, Sparks, MD] using conventional methods.[12] We reported culture tubes with no growth after 42 days incubation in the MGIT 960 instrument as culture-negative. We inspected cultures flagged as positive for the presence of AFB by ZN smear microscopy and sub-cultured them to blood agar plates to assess contamination. We discarded MGIT-positive, AFB-negative cultures with evidence of contamination. We re-incubated those without contamination in an auxiliary incubator for a total incubation time of 42 days, periodically re-examining them by ZN smear microscopy and blood agar cultures. We referred all MGIT-positive, AFB-positive cultures for identification of mycobacterial species. To salvage contaminated isolates, we re-decontaminated MGIT-positive, AFB-positive cultures and re-inoculated them into MGIT culture media.

#### Identification

We identified AFB-positive cultures as *Mycobacterium tuberculosis* complex (MTBC) using the Capilia TB Neo (Tauns Laboratories, Numazu, Japan) or MGIT TBc ID (Becton Dickinson, Sparks, MD) immunochromatographic assays. We tested all culture isolates that were AFB-positive but negative on the immunochromatographic assay for non-tuberculous mycobacteria (NTM) using the Genotype CM line probe assay (Hain Lifescience, Nehren, Germany).

### Definitions and Data Analysis

We regarded MGIT cultures flagged negative by the instrument at 42 days and culture isolates identified to have NTM (but not MTBC) as negative for MTBC. We regarded AFB-negative cultures with evidence of bacterial or fungal contamination as contaminated and not evaluable. We defined a TB case as any participant with laboratory-confirmed MTBC by at least one liquid culture test from any specimen or at least one Xpert MTB/RIF test if they had no previous history of TB treatment. We considered patients who had at least two sputum specimens negative for MTBC by Xpert MTB/RIF or liquid culture, and no positive result from any specimen, not to have TB. We excluded from analysis participants whose TB status could not be determined (i.e., those who did not have laboratory-confirmed MTBC and who did not have at least two specimens negative for MTBC). In comparisons with morning sputum, we regarded both spot sputum specimens as the same. For a given series of tests, we defined the incremental yield of each test in the series as the number and proportion of TB cases that were diagnosed using that test that were not diagnosed by the previous test(s) in the series, divided by the total number of TB cases that received all tests in the series. For calculations of incremental yield, we restricted analyses to cases that had all tests in the series and presupposed that patients would have a spot specimen collected before the morning specimen.

Data were captured electronically at the 15 clinical sites and in the laboratory and were analyzed using SAS version 9.3 (SAS Institute, Cary NC). We performed all calculations, including all proportions, as domain analyses, controlling for the complex design of the survey (i.e., clustering, stratification, weighting). Frequencies are presented as crude numbers, but proportions are based on weighted frequencies to account for the size of the clinic from which the patients were enrolled. Analyses incorporated the use of a finite population correction factor to account for the large sampling fraction. Chi-squared tests incorporated a Rao-Scott second order correction to account for the survey design.

### Ethical Considerations

All aspects of this study were approved by the Ethics Review Committee (ERC) of KEMRI (protocol number 1842) and by Institutional Review Board (IRB) G of the Human Resources Protection Office at the U.S. CDC (protocol number 5928). We requested a waiver of consent for testing of sputum specimens because: 1) the data and specimen collection, and corresponding test procedures, were not experimental (they were already recommended and used by the Kenya TB program); 2) the study activities posed no more than minimal risk to study participants; 3) participation did not adversely affect the welfare or rights of the patients in any way; and 4) to require formal written consent would have imposed an undue burden on the clinical staff of these busy clinics. Written informed consent was obtained for collection and testing of stool and lymph specimens, as these procedures were not routine or recommended at the time this study was conducted. The study protocols, including waiver of consent as specified above, were approved by the KEMRI ERC and CDC IRB with this methodology clearly described.

## Results

Final enrollment included 778 participants, from whom we collected 760 spot 1 sputa, 711 morning sputa, 738 spot 2 sputa, 283 stool and 17 LNA specimens; 692 participants submitted all three sputa, 61 submitted two, 11 submitted one and 14 submitted no specimens. Among the morning specimens collected, 49 had insufficient volume for Xpert MTB/RIF testing. The TB status of 38 participants could not be determined because of multiple contaminated or missing specimens, and we excluded them from further analyses.

Table 1 displays demographic and clinical characteristics of participants. MTBC was confirmed by liquid culture or Xpert MTB/RIF in specimens from 88 (11.3%; 95% confidence interval (CI): 10.0–12.6) participants, hereafter referred to as patients with TB; 85 had MTBC identified in sputum specimens, two had MTBC identified by stool culture only and one had MTBC identified by culture of LNA only. Thirty-three patients with TB had stool specimens cultured, 11 (33.3%; 95% CI: 9.5–57.1) of which were positive for MTBC; five patients with TB had LNA specimens cultured, all of which were positive. There were seven patients who were diagnosed by Xpert MTB/RIF alone, two of whom had contamination on both cultured sputum specimens, one of whom had one contaminated and one negative cultured sputum specimen, and four of whom had two negative cultured sputum specimens. One of them had a positive microscopy test, five were symptomatic and none had been previously diagnosed or treated for TB at any time in the past.

**Table 1:** Demographic and clinical characteristics of enrolled participants, stratified by tuberculosis (TB) diagnosis*.

	All Participants (n = 778); Column % (95% CI) or median (IQR) for continuous variables, as indicated†	Participants with TB (n = 88); Column % (95% CI) or median (IQR) for continuous variables, as indicated†	Participants without TB (n = 652); Column % (95% CI) or median (IQR) for continuous variables, as indicated†	p-value^‡^
Age in years, median (IQR)	29 (24–38)	31 (25–39)	29 (24–38)	0.67
Female sex (n = 514)	66.2 (62.0–70.3)	59.2 (52.1–66.3)	66.8 (62.6–71.1)	0.067
If female, pregnant^§^ (n = 141)	29.1 (20.8–37.3)	16.5 (6.5–26.4)	30.7 (22.4–38.9)	0.003
**District**				<0.0003
Kisumu (n = 182)	23.8 (0.0–47.5)	28.9 (2.1–55.6)	23.4 (0.0–46.9)	
Siaya (n = 292)	37.5 (17.2–57.7)	41.9 (19.3–64.5)	36.8 (16.6–57.0)	
Bondo (n = 207)	26.4 (6.6–46.2)	22.5 (4.1–41.0)	26.9 (7.0–46.8)	
Rarieda (n = 97)	12.3 (1.8–22.8)	6.7 (0.5–13.0)	12.9 (1.9–23.8)	
Lymphadenopathy reported**(n = 37)	4.7 (3.2–6.3)	9.6 (5.1–14.0)	4.4 (2.7–6.1)	0.074
Lymph node aspiration performed (n = 17)	2.2 (1.4–3.0)	5.7 (3.2–8.1)	1.8 (1.0–2.7)	<0.005
**CD4 count, cells/μl, median (IQR)**	343 (332–356)	159 (96–221)	360 (341–378)	<0.0001
Missing CD4 count (n = 64)	8.1 (6.6–9.7)	7.7 (3.1–12.4)	6.6 (5.1–8.0)	
CD4 count 0–99 (n = 114)	14.5 (12.6–16.5)	32.9 (27.7–38.1)	12.6 (10.1–15.1)	
CD4 count 100–199 (n = 88)	11.4 (9.9–12.8)	19.6 (13.5–25.7)	10.8 (9.2–12.3)	
CD4 count 200–499 (n = 318)	40.9 (39.3–42.5)	29.6 (22.7–36.6)	42.8 (41.3–44.3)	
CD4 count ≥500 (n = 195)	25.1 (23.2–27.0)	10.1 (6.6–13.7)	27.3 (25.2–29.3)	
**WHO stage of HIV disease**				<0.0001
Missing WHO stage (n = 26)	3.4 (1.5–5.3)	1.1 (0.0–22.5)	3.3 (1.3–5.3)	
I or II (n = 613)	78.7 (76.0–81.4)	47.7 (39.0–56.5)	83.1 (80.3–85.8)	
III or IV (n = 139)	17.9 (16.1–19.7)	51.2 (42.5–60.0)	13.7 (12.1–15.2)	
**Chest radiograph interpretation**				<0.0001
Missing chest radiograph (n = 193)	25.0 (18.6–31.4)	15.9 (9.7–22.2)	23.7 (16.7–30.8)	
Normal (n = 327)	41.9 (35.7–48.2)	19.3 (13.6–25.1)	46.2 (39.3–53.0)	
Abnormal (n = 258)	33.1 (25.7–40.5)	64.7 (56.3–73.2)	30.1 (22.1–38.1)	
Missing specific abnormal report (n = 5)	1.9 (0.0–4.3)	0	2.4 (0.0–5.6)	<0.0001
Abnormal, consistent with TB (n = 106)	41.2 (35.1–47.4)	71.9 (64.1–79.8)	31.6 (24.1–39.2)	
Abnormal, not consistent with TB (n = 147)	56.9 (50.7–63.2)	28.1 (20.2–35.9)	65.9 (58.1–73.7)	

* N = 778; one patient had no clinical or demographic data and is not represented here; TB status of 38 patients could not be evaluated

^†^ Percentages are weighted, and do not represent strict numerical proportions; 95% CI = 95% confidence interval, IQR = interquartile range

^‡^ p-values refer to comparison between ‘Patients with TB’ and ‘Participants without TB’; for comparisons of medians (Age and CD4 cell counts), p-values refer to Kruskal-Wallis test, for comparisons of categorical data (District and CD4 cell count categories), p-values refer to overall Chi-Square test.

^§^ Information on pregnancy status was missing for 22 female participants total: 1 excluded patient, 18 participants without TB, and 3 TB patients with TB

**Information about lymphadenopathy was missing for 34 participants total: 1 excluded patient, 29 participants without TB, and 4 patients with TB

### Comparison of morning and spot sputum specimens among patients with TB

Among patients with laboratory-confirmed TB, morning specimens appeared to be more sensitive than spot specimens for identification of MTBC by ZN microscopy, FM, and Xpert MTB/RIF (Table 2). Conversely, in liquid culture 69.2% of the morning specimens and 74.6% of spot specimens grew MTBC, while 10.7% and 7.1%, respectively, were contaminated (Table 2).

**Table 2:** Laboratory test results of participants diagnosed with TB, by type of test and specimen type (n = 88)*.

Specimen	ZN Result†		FM Result†		Xpert MTB/RIF Result†		MGIT Result†		
	Negative n (%; 95% CI)^§^	Positive n (%; 95% CI)^§^	Negative n (%; 95% CI)^§^	Positive n (%; 95% CI)^§^	Negative n (%; 95% CI)^§^	Positive n (%; 95% CI)^§^	Negative^‡^ n (%; 95% CI)^§^	Positive n (%; 95% CI)^§^	Contaminated n (%; 95% CI)^§^
**Spot sputum**	65 (77.6; 72.7–82.5)	19 (22.4; 17.5–27.32)	65 (74.8; 72.3–77.5)	22 (25.2; 22.5–27.8)	36 (43.0; 35.8–50.1)	48 (57.0; 49.9–64.2)	16 (18.3; 13.4–23.2)	65 (74.6; 69.3–79.9)	6 (7.1; 3.7–10.4)
**Morning sputum**	59 (69.5; 62.8–76.3)	26 (30.5; 23.7–37.2)	54 (63.7; 55.3–72.2)	31 (36.3; 27.8–44.7)	26 (34.0; 27.9–40.1)	50 (66.0; 59.9–72.1)	17 (20.0; 14.4–25.7)	59 (69.2; 61.4–77.0)	9 (10.7; 5.2–16.2)
**Lymph node aspirate**	n/a	n/a	n/a	n/a	n/a	n/a	0	5 (100, 100–100)	0
**Stool**	n/a	n/a	28 (84.8; 79.2–90.5)	5 (15.2; 9.5–20.8)	n/a	n/a	11 (33.3; 16.2–50.5)	11 (33.3; 9.5–57.1)	11 (33.3; 16.2–50.5)

* Of the 88 persons with TB, 84 submitted a spot sputum for ZN microscopy and Xpert MTB/RIF, 87 submitted a spot sputum for fluorescence microscopy and liquid culture, and 85 submitted a morning sputum for all tests; 9 of the morning specimens that were submitted by TB cases were not tested by Xpert MTB/RIF because of limited volume.

^†^ ZN = Ziehl-Neelsen microscopy; FM = Fluorescent Microscopy; Xpert MTB/RIF = GeneXpert MTB/RIF assay; MGIT = Mycobacterial Growth Indicator Tube

^‡^ Negative MGIT also includes non-tuberculous mycobacteria, which was identified on one spot 1 specimen.

^§^ Percentages are weighted, and do not equal the proportion based on unweighted counts; 95% CI = 95% confidence interval

### Diagnostic Yield

**Fig 2:**
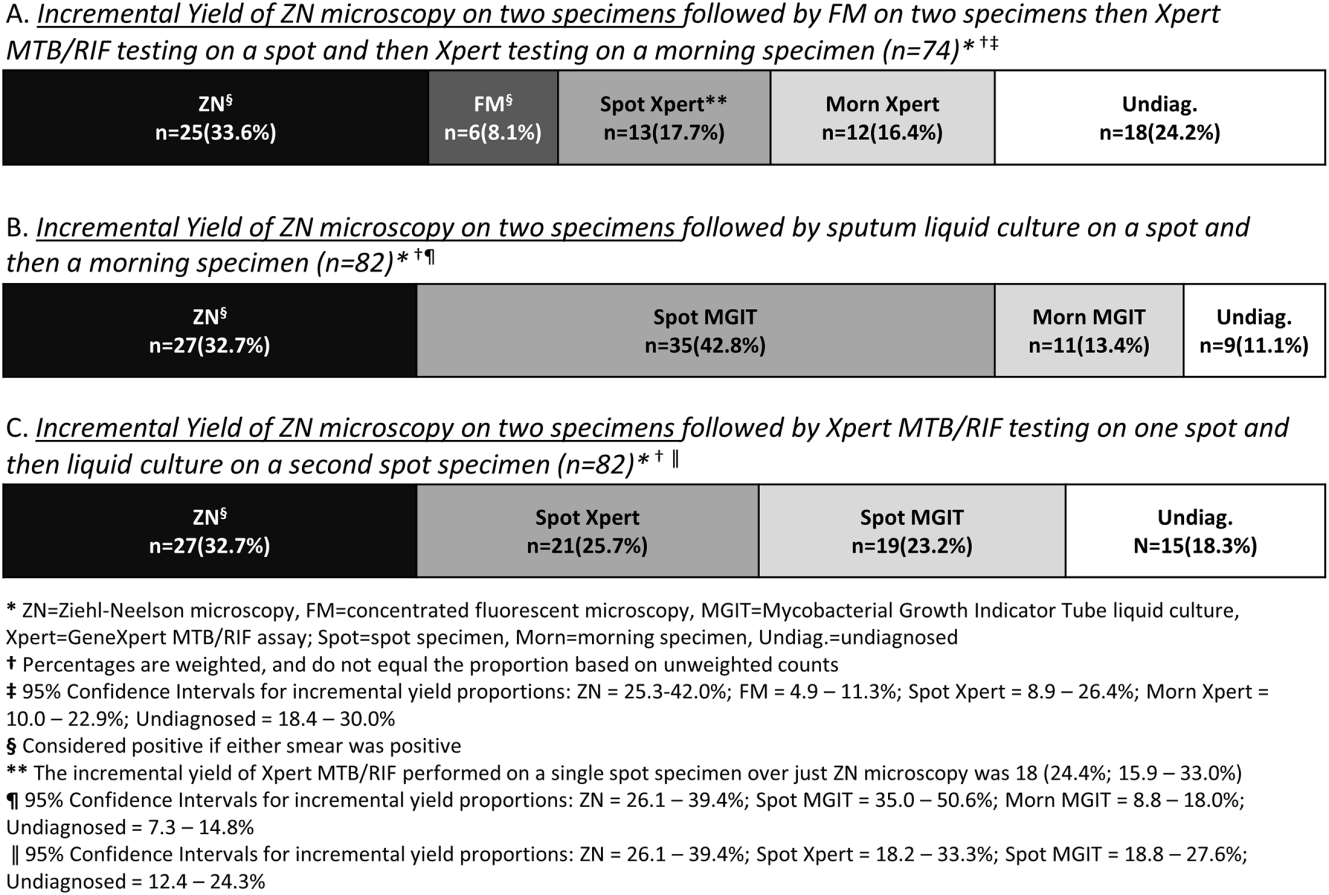
Incremental Yield of Different Diagnostic Tests.

Calculations of incremental yield require that all TB cases received all tests in the series; because patients with TB did not all receive the same tests, the cohorts from which the incremental yields were calculated differ in size. Fig 2 displays the incremental yield of different diagnostic tests performed on sputum specimens.

#### Diagnostic yield of Xpert MTB/RIF among patients with TB

Of 74 patients with TB who received Xpert MTB/RIF testing on one spot and one morning sputum specimen, 42 (56.7%; 95% CI: 49.8–63.6) were identified by testing a spot specimen and 49 (66.4%; 95% CI: 60.3–72.6) were identified by testing the morning specimen. Fourteen (19.1%; 95% CI: 13.9–24.4) additional cases were identified by testing the morning specimen after the spot specimen. Of the remaining 18 (24.2%; 95% CI: 18.4–30.0) patients not identified by Xpert MTB/RIF, three had MTBC cultured from both sputum cultures, nine had positive morning specimen cultures only, four had positive spot specimen cultures only, and one each had a positive stool and LNA specimen culture.

#### Diagnostic yield of liquid culture among patients with TB

Of the 84 patients with TB who provided one spot and one morning sputum specimen for liquid culture, 62 (73.7%; 95% CI: 68.5–78.8) were identified by culture of the spot specimen and 58 (68.9%; 95% CI: 61.1–76.7) were identified by culture of the morning specimen; 46 (54.6%; 95% CI: 47.0–62.1) were identified by both tests. Twelve (14.3%; 95% CI: 9.9–18.7) additional cases were diagnosed by culturing the morning specimen after the spot specimen. Of the 10 (12.0%; 95% CI: 8.6–15.4) patients not diagnosed by culture of two specimens, seven were identified by Xpert MTB/RIF testing of sputum, four of whom had two negative cultures and three of whom had one or two contaminated cultures. The three remaining patients had two negative sputum cultures and were identified by culture of an extra-pulmonary specimen: two patients had a positive stool culture and one patient had a positive LNA culture (Table 2).

### Comparison of ZN microscopy and FM, liquid culture, and Xpert MTB/RIF performed on morning specimen among patients with TB

Among the 76 patients with TB who had a morning sputum specimen tested by each method, 24 (31.5%; 95% CI: 23.4–39.6) were identified by ZN microscopy; FM would have identified all of those and an additional five (6.5%; 95% CI: 2.8–10.2). Xpert MTB/RIF identified all patients with a positive microscopy test and an additional 21 (28.0%; 95% CI: 16.6–39.3) smear-negative patients. Liquid culture identified an additional 10 (13.0%; 95% CI: 6.4–19.7) cases who were not identified by either microscopy or Xpert MTB/RIF, but Xpert MTB/RIF identified six patients who were not identified by liquid culture (two of whom were culture negative and four of whom had a contaminated culture). In total, liquid culture and Xpert MTB/RIF performed on a single morning specimen identified 60 (79.0%; 95% CI: 72.4–85.6) patients who had a morning sputum specimen tested by both methods (Table 2).

### Performance of Xpert MTB/RIF and liquid culture stratified by CD4 cell count among patients with TB

Compared to the composite definition, the sensitivities of Xpert MTB/RIF and liquid culture as individual tests differed by CD4 count and specimen type (morning vs spot). Xpert MTB/RIF, particularly with morning specimens, was more sensitive for patients with CD4 <100 cells/μL (Table 3).

**Table 3:** Sensitivity of Xpert MTB/RIF and liquid culture of sputum specimens provided by TB cases (as compared to a composite gold standard), stratified by CD4 cell count of participant and specimen collection type (spot vs. morning).

CD4 count*	Test	Overall sensitivity of 2 tests (by patient); n/N (%; 95% Conf. Int.)^†^	Specimen type	Sensitivity by specimen; n/N (%; 95% Conf. Int.)^†^
**CD4 < 100 cells/μL**	**Xpert**^‡^	22/24 (92.0%; 85.2–98.8%)	**Spot specimen**	18/28 (63.9%; 55.1–72.3%)
			**Morning specimen**	20/24 (83.6%; 76.0–91.2%)
**CD4 < 100 cells/μL**	**Liquid culture**^§^	25/29 (86.0%; 76.6–95.5%)	**Spot specimen**	19/29 (65.2%; 52.2–78.1%)
			**Morning specimen**	20/29 (68.5%; 58.2–78.8%)
**CD4 ≥ 100 cells/μL**	Xpert**	30/45 (67.0%; 59.1–74.9%)	**Spot specimen**	26/51 (51.2%; 41.7–60.7%)
			**Morning specimen**	25/46 (54.8%; 45.4–64.1%)
**CD4 ≥ 100 cells/μL**	**Liquid culture**^¶^	44/50 (88.0%; 83.4–92.6%)	**Spot specimen**	40/52 (77.0%; 71.6–82.4%)
			**Morning specimen**	33/50 (66.1%; 56.9–75.3%)

* 7 TB cases were lacking data on CD4 count;

^†^ Percentages are weighted, and do not equal the proportion of unweighted counts; 95% CI = 95% confidence interval

^‡^ 24 of 29 patients with CD4 count < 100 cells/μL had two Xpert MTB/RIF tests: five patients were missing an Xpert MTB/RIF report for morning specimen and one was missing an Xpert MTB/RIF report for both specimens;

^§^ 29 of 29 patients with CD4 count < 100 cells/μL had two liquid cultures

** 45 of 52 patients with CD4 count ≥ 100 cells/μL had two Xpert MTB/RIF tests: six patients were missing an Xpert MTB/RIF report for the morning specimen and one was missing a report for the spot specimen

^¶^ 50 of 52 patients with CD4 count ≥ 100 cells/μL had two liquid cultures: two patients were missing a liquid culture report for morning specimen

Initial CD4 counts were documented in 81 patients with TB. Of 24 patients who had CD4 count <100 cells/μL and who received two Xpert MTB/RIF tests, 16 (66.4%; 95% CI: 55.4–77.4) had a spot specimen that tested positive and 22 (83.6%; 95% CI: 76.0–91.2) had a morning specimen that tested positive; 6 (25.6%; 95% CI: 14.4–36.7) additional patients were diagnosed by testing the morning specimen after the spot specimen. Of the 45 (51.2%; 95% CI: 46.6–55.9) patients with TB who had a documented initial CD4 cell count ≥100 cells/μL and who received two Xpert MTB/RIF tests, 22 (49.2%; 95% CI: 38.9–59.4) had a spot specimen that tested positive and 25 (56.0%; 95% CI: 46.2–65.8) had a morning specimen that tested positive; eight (35.0%; 95% CI: 17.2–52.8) additional patients were diagnosed by testing the morning specimen after the spot specimen.

Of 1,471 cultured sputum specimens, 205 (13.9%; 95% CI: 12.3–15.5) were contaminated, including 86 (11.3%; 95% CI: 9.6–13.0) spot specimens and 119 (16.8%; 95% CI: 14.9–18.6) morning specimens. This compares to three (0.2%; 95 CI: 0.07–0.34) of 1,403 sputum specimens tested with Xpert MTB/RIF that had invalid results, all of which were spot specimens. Among specimens from patients with CD4 cell counts <100, 100–199, 200–499 and ≥500, cultures were contaminated, respectively, for 42 of 223 (19.1%; 95% CI: 14.9–23.2), 30 of 174 (17.1%; 95% CI: 10.5–23.6), 80 of 605 (13.2%; 95% CI: 11.4–15.0), and 35 of 371 (9.5%; 95% CI: 7.1–11.8).

## Discussion

With an incremental diagnostic yield of almost 18% over FM, and almost 25% over direct ZN smear microscopy, a single Xpert MTB/RIF greatly increased confirmation of TB disease, similar to what has been reported from other studies.[13] A second Xpert MTB/RIF diagnosed an additional 16% of TB patients not diagnosed by microscopy or a single Xpert MTB/RIF test. These data provide compelling programmatic evidence of the advantage of Xpert MTB/RIF over microscopy and again call into question the relevance of smear microscopy for TB diagnosis where Xpert MTB/RIF is available.[14, 15] Perhaps more notably, these data demonstrate how specimen type and origin impact test performance, which is directly relevant to TB programs struggling to determine the cost-benefit of these tests, and how best to maximize their performance and impact.

In our study, direct ZN microscopy detected only about one-third of TB cases confirmed by Xpert MTB/RIF or liquid culture, even though microscopy was performed in a research facility by well-trained technicians. Concentrated FM microscopy performed slightly better, approximating what has been reported in the literature.[16] Using ZN microscopy as an initial test for evaluating TB in PLHIV is not likely to be cost saving: in our study, very few of the 778 participants had a positive sputum smear, all others requiring investigation would have been referred for additional testing. Reliance on microscopy may even decrease the likelihood of treatment if clinicians are unaware of its remarkably limited sensitivity and assume that a negative test is meaningful, potentially increasing TB transmission and TB-related mortality.[17] As TB programs scale up newer molecular technologies,[18] replacement of microscopy with Xpert MTB/RIF may be more attractive, both clinically and financially, than reserving sequential testing with Xpert MTB/RIF for only those who test negative by smear microscopy, especially in settings of high HIV prevalence.[19–23]

Liquid culture is generally thought to be the most sensitive diagnostic technology, with a limit of detection that is generally under 10 organisms.[24] While this is true, especially in idealized laboratory settings, the overall practical performance (diagnostic yield) and utility of liquid culture are limited by specimen contamination and the laboratory efforts to limit it. We found that Xpert MTB/RIF had an overall diagnostic yield directly comparable to that of liquid culture, and performed better in persons with low CD4 counts compared to those with CD4 counts ≥100 cells/μl. Conversely, contamination of liquid culture increased as CD4 cell counts declined, reaching a frequency of 19% in cultured sputum specimens from patients with CD4 cell counts <100 cells/μL. These unevaluable results diminish the diagnostic yield of specimen culture, and partially explain why the sensitivity of culture did not increase with worsening immune compromise. It is generally considered that in PLHIV with lower CD4 cell counts, less cavitary disease leads to decreasing numbers of bacilli in sputum. However, our findings support the more recent hypothesis that those with the most severe immune compromise may suffer from unchecked, interstitial mycobacterial growth, which could cause a rebound in overall bacillary load to levels detectable by Xpert MTB/RIF, whether or not they are detected by microscopy.[13, 25–27]

Our data suggest that the performances of both liquid culture and Xpert MTB/RIF were also affected by specimen type (morning vs. spot). Previous research has demonstrated that the increased sensitivity of culturing a morning sputum specimen is offset by the increased contamination rate,[28, 29] and we found this to be true. In our analyses, approximately one in every seven cultured specimens was contaminated, and this contamination rate was higher for morning specimens than for spot specimens and, as stated above, higher for those with lower CD4 cell counts. This proportion of contamination is similar to that seen in other programmatic laboratories in resource-limited settings,[30–32] and is an important limitation for culture-based diagnostic approaches. Importantly, this trade-off does not extend to Xpert MTB/RIF, which, as a molecular test, is largely unaffected by the presence of contaminating fungus or bacteria. While this finding was not statistically significant, our data show a trend towards higher diagnostic yield of morning specimens than spot specimens when tested by Xpert MTB/RIF; this increased when the morning specimens were from patients with CD4 cell counts <100 cells/μL.

It is substantially easier to implement automated genetic testing, such as Xpert MTB/RIF, than to implement liquid culture, which is technically demanding and expensive, and the global scale-up of Xpert MTB/RIF is certainly outpacing that of liquid culture. The debate about which test has the higher diagnostic yield may soon be obviated, however, by a new MTB/RIF cartridge for the GeneXpert machine, which has been reported to have a limit of detection comparable to liquid culture. (http://www.croiconference.org/sessions/xpert-mtbrif-ultra-new-near-patient-tb-test-sensitivity-equal-culture) When those cartridges are available and have been validated in program settings, they may prove to have a significantly higher diagnostic yield than culture in all circumstances. But culture-based testing does allow clinicians to distinguish between active TB disease and previously treated disease, and Xpert MTB/RIF does not. Moreover, culture-based testing allows for subsequent drug susceptibility determination and refinement of treatment for patients with drug-resistant disease; in circumstances where drug resistance is an issue and an indication for testing, culturing of specimens is a top priority. As each test offers different benefits, TB diagnosis would undoubtedly be maximized by utilizing both tests, and interpreting results in the correct clinical context, which would increase case-finding and reduce transmission. Programs must realistically assess their own capabilities and implement the best sequences of testing to address their needs.

Globally, TB programs have been incorporating Xpert MTB/RIF into screening and diagnostic algorithms, but are struggling to determine if and under what circumstances it should be repeated if an initial test is negative. Our data suggest that the cumulative sensitivity of 2 Xpert MTB/RIF tests is higher in those with a CD4 count <100 cells/μL (92%) than in those with CD4 count ≥100 cells/μL (67%). Even in resource-limited settings, it may prove cost-effective to repeat Xpert MTB/RIF testing for PLHIV with CD4 counts <100 cells/μL who are at the highest risk for both TB disease and TB-related early mortality. [33]

Lymphadenopathy in our study population may have been underreported, as rates were lower than reported elsewhere, and aspiration was rarely performed.[34, 35] Importantly, however, MTBC was identified by LNA culture in 29% of participants who had an aspiration and in all TB cases for which LNAs were cultured. This adds to other literature on the impact of this diagnostic technique and provides rationale to increase programmatic capacity and utilization in the correct clinical circumstance (i.e., in patients with apparent enlarged lymphadenopathy whose clinical presentation suggests TB).[34, 36, 37]

This study has several limitations. Our ability to compare same-specimen test results was limited by the testing algorithm in our study, which apart from the morning specimen, assigned different tests to different specimens. Because Xpert MTB/RIF testing of morning specimens was restricted by volume in our protocol, it may be that analyses of the morning specimen results were biased. We think this is unlikely, as we found no association between sputum volume and culture result for the morning specimen, nor did we find an association between Xpert MTB/RIF result and sputum volume for spot specimens. We also assumed that the yield for spot 1 and spot 2 sputum specimens was the same. We included as cases patients who were identified by Xpert MTB/RIF alone. Given reports of false-positive Xpert MT/RIF, this may seem somewhat controversial. The most likely causes of discordance between Xpert MT/RIF and culture include the presence of dead bacteria, leading to false-positive Xpert results, and the consequences of specimen decontamination, which can render mycobacteria non-viable, leading to false-negative culture results. None of the patients in this study who had discordant results were previously treated for TB, and most of them were symptomatic. Given the prevalence of TB in this population, and the known limitations of culture, we felt it appropriate to include them as cases. Because we were unable to confirm clinical characteristics for all participating patients, we could not determine the true prevalence of lymphadenopathy, which was lower than expected. This also limits what we can say about the use of LNA for TB diagnosis. However, our use of multiple specimens for Xpert MTB/RIF and culture diminish the possibility that we missed cases of active TB disease.

Sputum smear microscopy has long been the most widely used method for diagnosing TB, but has limited sensitivity, and using it in parallel with more sensitive technologies is unlikely to be worthwhile.[38] A single Xpert MTB/RIF is a better diagnostic test, and its sensitivity may be further enhanced by targeted testing and using a morning specimen, a consideration for programs unable to use it as an initial test for all symptomatic patients. It may be prudent to offer a second Xpert MTB/RIF for the most immune compromised patients, who would derive the biggest benefit and for whom the test is most sensitive. Additionally, sensitivity is expected to improve as the next generation of cartridges, Xpert MTB/RIF Ultra, are introduced into program settings; evaluating the impact of specimen type and CD4 cell count on the performance of these new cartridges should be an important early investigation that could directly inform program policy. Research to determine strategies to further investigate symptomatic PLHIV that test negative by Xpert MTB/RIF, and those that will test negative by Xpert MTB/RIF Ultra, is urgently needed.
